# Influence of Water Storage on the Mechanical Properties of 3D-Printed Aligners: An In Vitro Study

**DOI:** 10.3390/bioengineering13010021

**Published:** 2025-12-26

**Authors:** Kathrin Puchert, Paul Ritzert, Sebastian Wille, Jusef Naim, Sinan Şen

**Affiliations:** 1 Department of Orthodontics, University Hospital Schleswig-Holstein, Campus Kiel, 24105 Kiel, Germany; 2 Department of Dental Prosthetics, University Hospital Schleswig-Holstein, Campus Kiel, 24105 Kiel, Germany

**Keywords:** direct printed aligner, mechanical properties, water storage

## Abstract

Directly printed aligners represent a promising alternative to conventional thermoformed aligners. The aim of this in vitro study was to compare the effects of water on the mechanical properties of directly printed aligners with those of conventionally manufactured thermoformed PET-G foils. Dental LT Clear V2 (LT), V Print Splint Comfort (VP), and TC-85 DAC (TC) were examined. Biolon (BL), a conventional PET-G material, served as the thermoplastic reference material. All samples were tested before and after 14 days of water storage at 37 °C. We performed a three-point bending test and an indentation test, and examined changes in the abrasion resistance and hygroscopic volume. The resistance of all printed specimens decreased significantly after water storage. VP and TC were less resilient than BL overall. LT and BL exhibited the lowest indentation creep (BL: 0.08 ± 0.01, LT: 0.13 ± 0.02, VP: 0.21 ± 0.02, TC: 0.24 ± 0.02). Furthermore, the abrasion of LT (0.72 ± 0.21 mm^3^) was significantly lower than that of BL (1.12 ± 0.37 mm^3^). In conclusion, the water sorption of the printed test specimens had a significant influence on the mechanical properties, with a reduction in the flexural modulus, Martens hardness, and plastic hardness.

## 1. Introduction

The introduction of the first nearly invisible aligners in the United States in 1999 marked a significant turning point in orthodontics. At that time, the profound impact this technology would have could not be foreseen. The discreet tooth correction offered by aligners provides numerous advantages, including improved aesthetics, increased comfort, and the ability to maintain oral hygiene during treatment [1,2,3,4]. These advantages triggered a wave of innovation, with numerous companies offering aligner systems on the market. However, aligner therapy also presents certain challenges. For example, they are limited in treating more complex orthodontic cases, and traditional fixed appliances are often superior in these situations [5,6]. The success and effectiveness of aligner therapy depends on the mechanical properties of the aligner material, as these significantly influence the transmission of force to the teeth and thus the effectiveness of tooth movement during treatment [7,8].

Aligners produced using the thermoforming process are the established standard in orthodontic treatment. In this method, thermoplastic sheets are heated and vacuum-formed over a dental model to create a precisely fitting appliance. Polyethylene terephthalate glycol (PET-G) is typically used for this because of its high strength, good transparency, and excellent deformability when heated [9,10,11].

Despite these advantages, PET-G also has limitations. Under continuous mechanical stress, it undergoes time-dependent indentation creep and material fatigue, which can lead to a gradual loss of the forces transmitted to the teeth [11,12,13]. In addition, PET-G has limited abrasion and chemical resistance. Contact with saliva, food, or cleaning agents can cause surface changes, cloudiness, or discoloration, which can negatively affect both aesthetics and hygiene [14,15]. Studies have also shown that PET-G can develop plastic deformations under repeated bending stress, leading to reduced effectiveness of tooth movement [13,16].

Due to these limitations and the increasing demand for more individualized and efficient orthodontic solutions, focus has been placed on the development of alternative manufacturing methods and materials. Recent advances in additive manufacturing, particularly in 3D printing of aligners, have opened new possibilities to improve the mechanical properties of aligner materials while simultaneously allowing treatment to be customized. The geometry of printed aligners can be precisely controlled, for example through targeted variation of wall thickness or adjustment of edge design. This can optimize not only force distribution but also wearing comfort [17,18,19,20].

There are two additive printing processes: stereolithography (SLA) and digital light processing (DLP). In SLA, a UV laser scans each layer point by point and polymerizes it, whereas a DLP printer works with a digital projector that can expose an entire layer at once. These printed aligners have shown superior material properties compared with thermoformed aligners. Shirey et al. demonstrated significant differences in the elastic modulus, tensile strength, and stress relaxation between 3D-printed and thermoformed aligners. However, moisture in the oral cavity influenced the mechanical properties of 3D-printed aligners more strongly than those of thermoformed aligners. This may impair the ability of printed aligners to generate and maintain a consistent and sufficient force for tooth movement [21].

To investigate this potential issue further, the aim of this in vitro study was to compare the effects of water on the mechanical properties of directly printed aligners with those of conventionally manufactured thermoformed PET-G foils. Specifically, we measured water storage, Martens hardness, plastic hardness, indentation creep, abrasion resistance, and water absorption. These results help assess the clinical potential of modern aligner technologies and provide an evidence-based foundation for selecting suitable materials in orthodontic therapy.

## 2. Materials and Methods

### 2.1. Choice of Materials

We compared a conventional thermoplastic aligner material with three 3D printed aligner materials (one made by SLA and two made by DLP). The thermoplastic material was Biolon (BL) and served as our reference material. The SLA-printed aligner was Dental LT Clear V2 (LT), and the two DLP-printed aligners were V Print Splint Comfort (VP) and TC-85 DAC (TC). Details of these materials are presented in Table 1.

### 2.2. Production and Post-Processing of the Test Specimens

**Figure 1:**
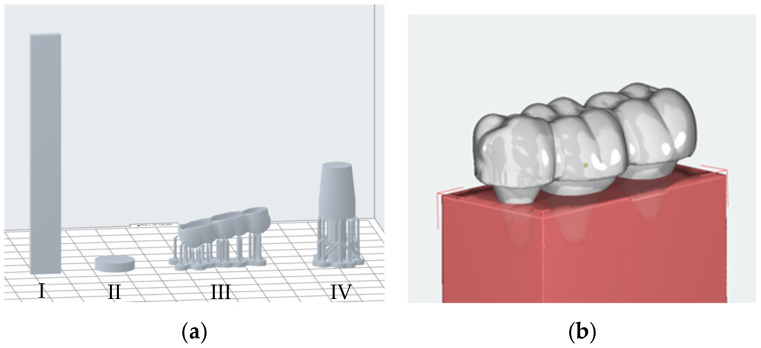
(**a**) Printing preview (Preform, Formlabs) of the test specimens: three-point bending test (**I**), indentation test (**II**), abrasion resistance (**III**), hygroscopic volume increase (**IV**); (**b**) digital design of the splint sections on the typodontic model (Onyxceph, Image Instruments).

The thermoplastic BL (Dreve Dentamid, Unna, Germany) was thermoformed onto a flat plaster disk and then onto a printed section (Model V2, Formlabs, Somerville, MA, USA) of a typodontic model (KaVo Dental, Biberach, Germany) of teeth 18 to 20. After this, it was cut to size for the respective tests. The orientation of the printed specimens is shown in Figure 1a. The print orientation for the three-point bending test specimens and for the examination of the hygroscopic volume increase ensured that the height and width were consistent among different samples. The splint segments for the abrasion resistance testing were oriented in a similar fashion as an aligner would be.

The bending specimens (height: 3 mm, width: 10 mm, length: 80 mm) were manufactured 15 times. The test specimens were not subjected to any further processing beyond what is required for the printing and post-curing process. Unlike the method shown in Figure 1a, support structures had to be used to print the bending specimens with TC at the instruction of the distributor Forestadent (Pforzheim, Germany), who is an official partner of Graphy.

The disks for the indentation test (height: 3 mm, diameter: 13 mm) were manufactured 15 times. For this test, all test specimens of the various materials were finished using P1200 grained sandpaper to ensure a consistent arithmetic average roughness (R_a_). In addition, laser microscopy (VK X-100 Series, Keyence, Neu-Isenburg, Germany) was used to ensure that the R_a_ value was below 5% of the indentation depth, in accordance with the specifications of DIN 14577. The measured R_a_ values were consistently below 0.5 µm.

The splint sections (thickness: 0.75 mm) were fabricated eight times. In addition, a typodontic model was converted into an STL file using an intraoral scanner (Trios 4, 3Shape, Copenhagen, Denmark) and digitally processed (Meshmixer, Autodesk, San Rafael, CA, USA) so that only the models of teeth 18 to 20 were anchored in a rectangular block. The STL file of the model (Figure 1b) was exported (Onyxceph, Image Instruments, Chemnitz, Germany), converted (PreForm, Formlabs), and printed (Model V2, Formlabs) using a Form 3B+ Printer (Formlabs). Rubber polishers were then used to remove residues of printing supports, and the desired finish of all different material groups was achieved using a polishing machine. Quality assurance was carried out using laser microscopy, as with the test specimens for the indentation test. The specimens were then adhesively bonded to the models to prevent loosening during the chewing simulation.

The truncated cones for determining the increase in hygroscopic volume (H = 11.7 mm; r_1_ = 9.5 mm; r_2_ = 12 mm) were manufactured eight times. No further post-processing was carried out beyond that of the printing and post-curing process.

LT (Formlabs) was produced using an SLA printer (Form 3B+, Formlabs). The test specimens were then washed for 15 min with 99% isopropanol (Form Wash, Formlabs), air-dried, and post-cured for 60 min (Form Cure, Formlabs) in accordance with the manufacturer’s specifications.

The VP (VOCO) test specimens were produced using DLP. The test specimens were then cleaned for 8 min in an ultrasonic bath with 99% isopropanol. The post-curing comprised 2 × 2000 flashes (Otoflash G171, Dentona, Dortmund, Germany).

TC (Graphy) test specimens were also manufactured by DLP. After printing a centrifuge (FO 415-0003, Graphy) was used for 6 min to eliminate excess resin. Post-curing was performed using a nitrogen curing machine (Terra Harz Cure, Graphy) for 20 min.

TC and VP were manufactured and supplied directly by the corresponding manufacturers who stated that they had complied with official guidelines. The same printing instructions were provided to the manufacturers that were used for LT. However, it was not possible to use the same layer thickness (VP: 0.05 mm, LT: 0.1 mm, TC: 0.1 mm) due to closed system restrictions. Before testing all specimens were measured using a caliber. Only the ones within the ranges of the correlating norms were used. DIN 178 applies to the 3-point bending test with tolerable deviations of ±2 mm in in length and ±0.2 mm in width and height [22]. DIN 14577 applies to the indentation test, which stipulates that the test specimen should be 10 times thicker than the indentation depth [23].

### 2.3. Water Storage

Before testing, specimens were stored for two weeks in distilled water at 37 °C. The aged specimens were exposed to laboratory conditions (22 ± 2 °C and 45 ± 5% humidity) for 3 h before testing in accordance with the suggested conditioning by DIN 23529 [24]. This conditioning served as a baseline for the comparison of the mechanical properties due to aging processes before and after aging regardless of environmental factors. In addition, all specimens were stored in a dark container in a suitably temperature-controlled room prior to testing.

### 2.4. Testing

#### 2.4.1. Experiment Implementation of the Three-Point Bending Test (DIN 178)

**Figure 2:**
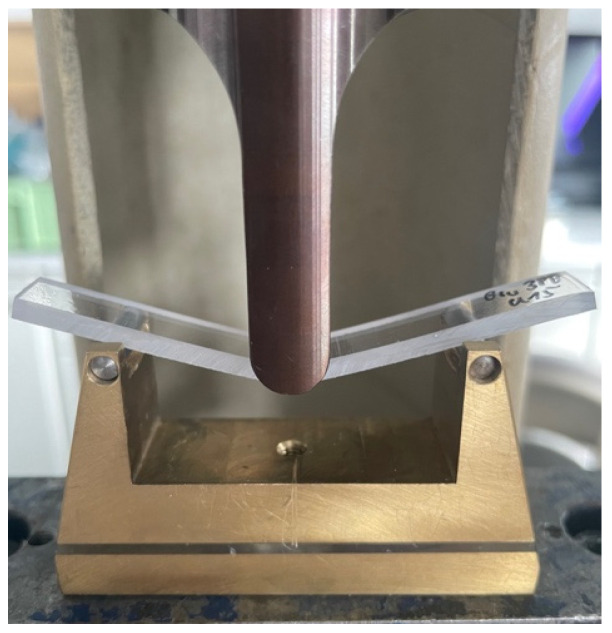
Experimental setup for the three-point bending test.

Test specimens were placed on rods with a radius of 2 mm, which were arranged at a distance of 50 mm from each other (Figure 2). The preload was set to 0.5 N. A fin with a radius of 5 mm was mounted in a universal testing machine (Z010, ZwickRoell, Ulm, Germany) and pressed onto the center of the specimens at a speed of 2 mm/min. The largest possible deflection of 15 mm was selected as the end point in this test setup.

Force and deflection were recorded continuously so that a force–displacement curve could be created (Testexpert, ZwickRoell). The flexural modulus was then calculated from the raw data as follows [22]:

$$E_{f}=\frac{\sigma_{f2}-\sigma_{f1}}{0.0025-0.0005}$$

$E_{f}$ = flexural modulus [MPa]
$\sigma_{f1}$ = bending stress at 0.25% bending strain [MPa]
$\sigma_{f2}$ = bending stress at 0.05% bending strain [MPa]

#### 2.4.2. Experiment Implementation of the Indentation Test (DIN 14577)

**Figure 3:**
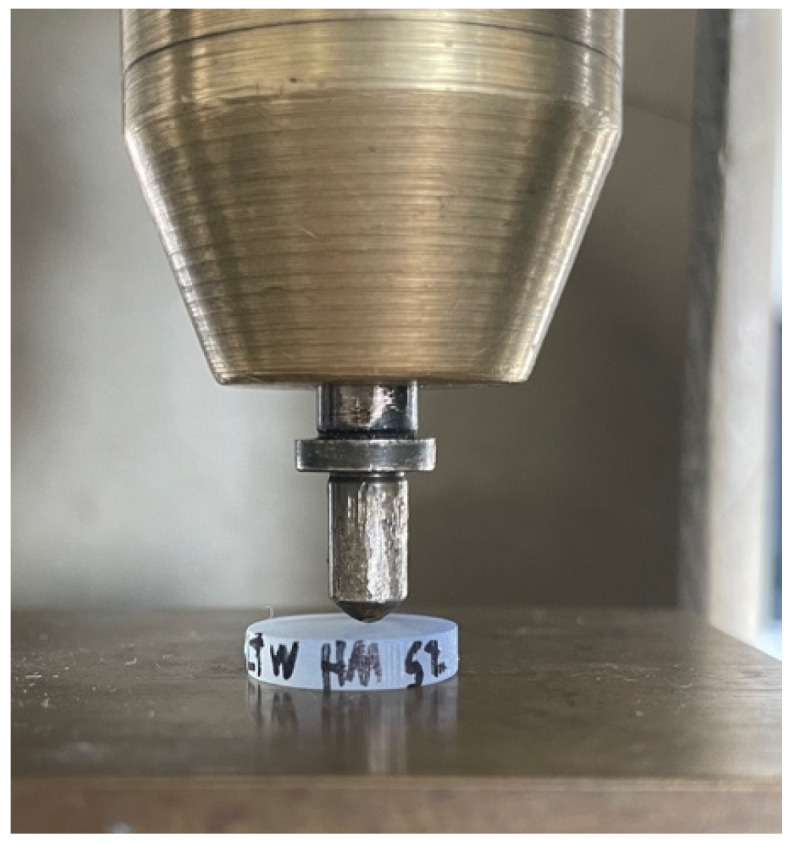
Experimental setup for the indentation test.

A Vickers diamond was mounted as an indenter in a universal testing machine (Z010, ZwickRoell). The preload was set to 0.25 N. Once this value was exceeded, a force of 6 N was applied at a speed of 0.2 µm/s to test in the macro range [23]. The maximum force was then held for 30 s. Finally, the indenter retracted at a speed of 0.2 µm/s (Figure 3).

Force and indentation depth were recorded continuously so that a force–displacement curve could be created (Testexpert, ZwickRoell). The Martens hardness, the plastic hardness, and the indentation creep were then calculated from the raw data as follows [23]:

$$HM=\frac{F_{max}}{26.43*h^{2}}$$

HM = Martens hardness [N/mm^2^]
F_max_ = maximum force [N]
h = penetration depth [mm]

$$H_{plast}=\frac{F_{max}}{26.43*{h_{r}}^{2}}$$

H_plast_ = plastic hardness [N/mm^2^]
F_max_ = maximum force [N]
h_r_ = intersection of the tangent to the unloading curve at Fmax with the depth axis [mm]

$$C_{IT}=\frac{h_{2}-h_{1}}{h_{1}}$$

C_IT_ = indentation creep [ ]
h_1_ = indentation depth at the beginning of the holding period [mm]
h_2_ = indentation depth at the end of the holding period [mm]

#### 2.4.3. Experiment Implementation of Abrasion Resistance

The models and splint sections were fixed in a chewing simulator (CS4, Feldkirchen, SD Mechatronic, Germany). A steatite ball with a diameter of 6 mm was selected as the antagonist. During the chewing cycles, the steatite ball impacted centrally on the occlusal surface of the splint sections around tooth 19. The chewing simulation consisted of a cycle of 10,000 chewing movements at a frequency of 1.3 Hz and a laterotrusion movement of 0.5 mm under a load of 50 N. In addition, a thermal cycle was maintained with alternating 5 °C and 55 °C distilled water. The testing conditions were chosen to simulate long-term use.

Before and after the chewing simulation, the topography of the occlusal surface of the splint sections were recorded using a laser microscope (VK X-100 Series, Keyence). An anti-glare spray was applied beforehand to minimize artifacts caused by reflections. The data of the occlusal surfaces before and after the chewing simulation were superimposed using a best-fit function (Geomagicwrap, Oqton, San Francisco, CA, USA). Three-dimensional bodies were calculated from the deviating surfaces created by abrasion. The volume of these bodies was equivalent to the respective abrasion.

#### 2.4.4. Experiment Implementation of the Hygroscopic Volume Increase

**Figure 4:**
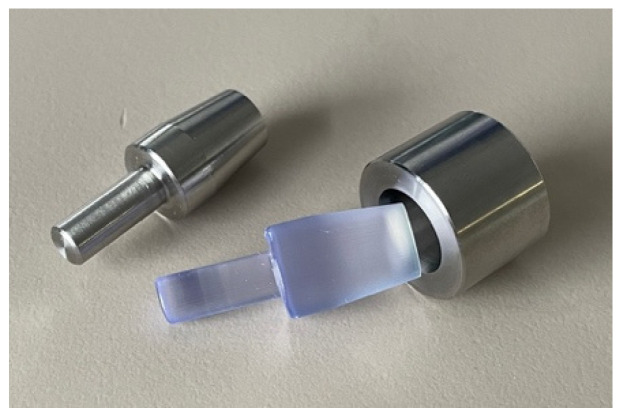
Milled and printed parts for determining the hygroscopic volume increase.

The reference body could not be manufactured from BL in this experiment, so a computerized numerical control (CNC)-milled reference body was used as described in Section 2.2. This was manufactured from titanium using a CNC milling machine (Traub TNS26, Index-Werke, Esslingen am Neckar). A counterpart for the converging surfaces was also milled (Figure 4).

All test specimens were first measured in a dry state and compared with the reference test specimen before recording the deviations in volume from the reference specimen. The printed truncated cones were then stored in distilled water at 37 °C for two weeks. After this time, the test specimens were brought to room temperature (22 ± 2 °C) in distilled water for three hours and wiped dry before a second measurement. In this measurement, the increase in hygroscopic volume of the sample was determined and compared with that of the milled reference specimen.

**Figure 5:**
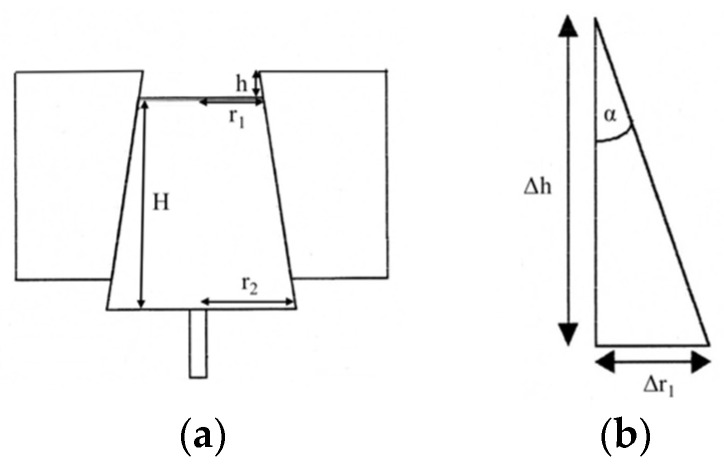
(**a**) Schematic representation of the measurement of hygroscopic volume increase and (**b**) the trigonometric background.

A caliper was used to measure the distance between the tip of the truncated cone and the edge of the counterpart. This distance was used to calculate the radius of the tip of the truncated cone and thus the volume of the body (Figure 5). The calculations were performed as follows:

$${\Delta r}_{1}=\tan\left(6^{{}^{\circ}}\right)*\Delta h$$

${\Delta r}_{1}$ = change in the radius of the upper surface [mm]
Δh = change in the measured distance [mm]

$$V=\frac{1}{3}*\pi *H*({r_{1}}^{2}+r_{1}*r_{2}+{r_{2}}^{2})$$

V = volume of a truncated cone [mm^3^]
H = height [mm]
r_1_ = upper radius [mm]
r_2_ = lower radius [mm]

### 2.5. Statistical Analysis

SPSS (Version 27, IBM Corp., Armonk, NY, USA) was used for statistical analysis of the results. All results are presented as mean values and standard deviations. Statistical significance was defined as *p* < 0.05.

The Levene test revealed no homogeneity of variances for the Martens hardness and plastic hardness measurements (*p* < 0.001), so these parameters were analyzed using one-way ANOVA followed by the Games–Howell post hoc test. For the indentation creep and flexural modulus measurements, two-way ANOVA revealed statistically significant interactions between the material type and water storage (*p* < 0.001), so additional one-way ANOVA and Tukey post hoc tests were also applied. The statistical significance of the difference in abrasion volume was determined using one-way ANOVA. Only two materials could be properly statistically evaluated, so no post hoc test was necessary. The Shapiro–Wilk test revealed that the hygroscopic volume data were not normally distributed (*p* < 0.05), so these data were assessed using the Kruskal–Wallis test.

## 3. Results

### 3.1. Three-Point Bending Test/Flexural Modulus

**Figure 6:**
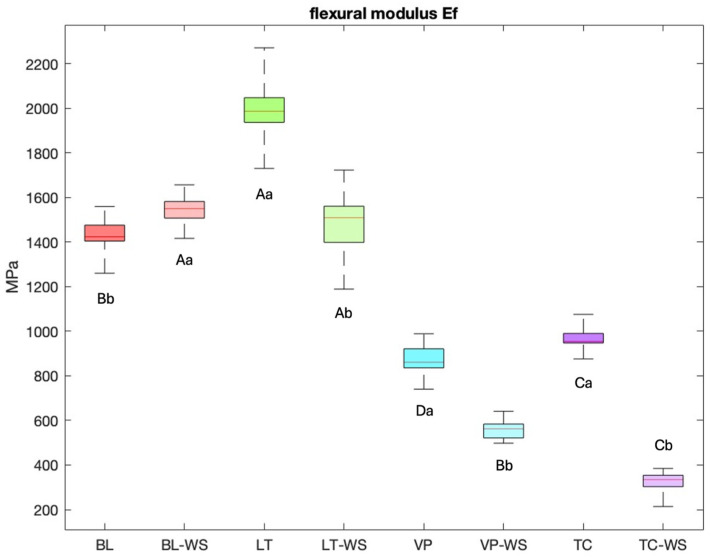
Results of the three-point bending test. WS = testing after water storage; different capital letters indicate statistically significant differences (*p* < 0.05) between different materials within the same water storage condition; different lower-case letters indicate statistically significant differences (*p* < 0.05) between different water storage condition of the same material.

The flexural modulus was determined using the three-point bending test. In the dry state, the flexural modulus of LT (2000 ± 131 MPa) was significantly higher than that of the thermoplastic BL (1429 ± 77 MPa) (Figure 6). However, VP (866 ± 70 MPa) and TC (967 ± 49 MPa) had a significantly lower flexural modulus than BL and LT did. After storage in water, the flexural modulus of all 3D-printed specimens decreased significantly (LT: 1485 ± 153 MPa, VP: 557 ± 38 MPa, TC: 320 ± 47 MPa). In contrast, the flexural modulus of BL actually increased significantly (1543 ± 67 MPa). It is also noteworthy that the flexural modulus of LT did not differ significantly from that of BL after water storage.

### 3.2. Indentation Test

All printed test specimens exhibited a lower resistance behavior after water storage than before (Table 2). In contrast, no significant reductions were observed in the resistance behavior of the thermoplastic BL. Furthermore, VP and TC were significantly softer and easier to deform than BL and LT, especially after storage in water.

### 3.3. Abrasion Resistance

**Figure 7:**
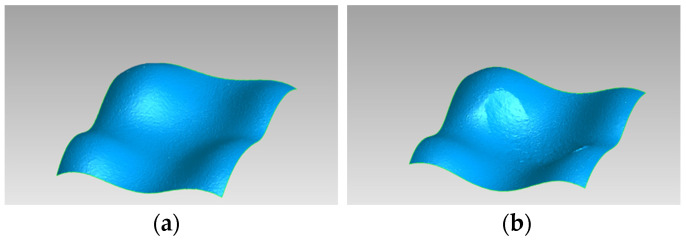
Laser microscopy of the occlusal surfaces (BL, Dentamid) (**a**) before and (**b**) after the chewing simulation (Geomagicwrap, Oqton).

Due to cracking, the function of the softer materials failed in several splint sections (TC: 5 of 8; VP: 3 of 8). The abraded volume of TC (2.01 ± 0.40 mm^3^) and VP (1.05 ± 0.23 mm^3^) could not be evaluated statistically. The abrasion of LT (0.72 ± 0.21 mm^3^) was significantly lower than that of BL (1.12 ± 0.37 mm^3^) (Figure 7).

### 3.4. Hygroscopic Volume Increase

The volume of LT relative to the milled reference body before water storage was significantly higher than that of VP and TC (Table 3). However, there were no significant differences in the increase of hygroscopic volume between the different materials.

## 4. Discussion

This in vitro study systematically evaluated how water affects the mechanical properties of 3D-printed aligner materials compared with a thermoformed reference material. Mechanical parameters were determined via three-point bending tests and indentation measurements, and physical–functional properties were assessed based on their abrasion resistance. In all tests, water absorption was considered an aging process to realistically simulate the clinical conditions—particularly the continuous exposure to saliva in the oral environment. Based on these results, the suitability of the materials for use in aligner therapy can be differentially assessed.

### 4.1. Three-Point Bending Test

The elastic modulus represents a key material parameter for characterizing elastic deformation behavior and provides insights into an aligner material’s ability to transmit controlled orthodontic forces to the teeth under functional loading. In this study, the flexural modulus of the 3D-printed materials was determined using a three-point bending test, both in dry conditions and after water storage, to quantify flexural stiffness under conditions that closely simulate the intraoral environment.

The flexural modulus differed markedly between the 3D-printed aligner materials, both prior to and following immersion in distilled water at 37 °C. Before water storage, LT exhibited the highest flexural modulus, indicating greater stiffness, whereas VP and TC demonstrated significantly lower values. Notably, all additively manufactured materials showed a distinct reduction in flexural modulus after water storage. As reported by Shirey et al., this decline is likely attributable to pronounced water sorption, potentially resulting from incomplete polymerization during the additive manufacturing and post-curing process. This insufficient crosslinking facilitates the diffusion of water molecules into the polymer network, which can disrupt intermolecular interactions and compromise mechanical integrity [21]. Consistent with these findings, Berli et al. reported significantly higher water uptake in 3D-printed occlusal splints than in pressed or milled counterparts, suggesting an inherently higher hygroscopicity of printed resins [25].

In contrast, the flexural modulus increased in the thermoformed reference material following water storage. This can be attributed to the physical post-curing processes within the PET-G polymer network. Specifically, the moderate thermal exposure in combination with the limited but present water absorption may facilitate cold crystallization within initially amorphous regions of the material by loosening polymer chain structures. This process promotes denser molecular packing, thereby increasing material stiffness [26,27,28,29].

Interestingly, after water storage, the flexural modulus of the LT material closely approached that of the BL reference material. This convergence toward a clinically established standard is favorable because it indicates that the 3D-printed material is suitable for practical application. At the same time, the observed decrease in stiffness after water storage suggests that intentional pre-soaking prior to clinical use may reduce the initial high material rigidity. A moderately reduced stiffness can significantly improve intraoral handling, particularly during insertion and removal of the aligner. In addition, aligner materials with a higher flexural modulus can be produced significantly thinner at the same force [11], making the design more flexible and opening up new possibilities for the individual customization of printed aligners. Jindal et al. also compared 3D-printed LT aligners to conventionally thermoformed aligners, and found that the 3D-printed variants demonstrated higher mechanical resilience and lower deformation, indicating that they may represent a viable alternative to thermoformed aligners [20].

The TC material exhibited the lowest flexural modulus after water storage, indicating the highest flexibility among the tested materials. This property may enhance wearing comfort, soft tissue compatibility, and individualized adaptability. However, such soft materials are generally associated with reduced mechanical efficacy, particularly regarding the controlled transmission of orthodontically effective forces.

TC-85 is characterized by its pronounced shape-memory effect and high flexibility. Lee et al. found that 3D-printed aligners fabricated from Tera Harz TC-85 demonstrated greater flexibility and a wider elastic range than thermoformed PET-G aligners. They also showed greater tooth movement per aligner and the application of consistent orthodontic forces to the teeth without a reduction in force due to aligner deformation [30].

### 4.2. Indentation Test

Martens hardness is a characteristic value for the mechanical resistance of a material to the penetration of a test body. It describes how resistant the aligner material is to deformation. A high value means that the aligner remains dimensionally stable and the forces exerted on the tooth are reliably transferred. According to Alexandropulos et al., a high Martens hardness is advantageous [11]. If the Martens hardness is very low, the aligner yields slightly under load and deforms, which means forces cannot be transferred effectively to the tooth. To achieve realistic results, the tests were repeated after two weeks of storage in water at 37 °C to simulate the intraoral saliva storage of the aligners.

The LT material showed the highest Martens hardness among the aligner materials tested, although this value decreased after storage in water. The Martens hardness of the BL standard changed only slightly after storage in water. It remained dimensionally stable even under water-containing conditions. These high Martens hardness values of LT aligners under dry conditions indicate higher initial hardness and possible improved force transmission during the intraoral wearing period, approaching the value of the thermoformed BL reference material.

In contrast, Martens hardness values were significantly lower in the DLP-printed materials (VP, TC) than the reference material (BL). These values decreased even further after water storage, indicating that these materials lose dimensional stability when exposed to moisture, such as in the intraoral environment. This may impair effective transfer of force to the teeth.

Plastic hardness was measured in the specimens to determine their resistance to permanent deformation when placed under stress. This parameter is particularly important for aligners because, with insufficient plastic hardness, they can lose their dimensional stability over time, a decrease that can be further exacerbated by the stresses of the oral environment. This impairs the precise transfer of force to the teeth and may cause planned tooth movements to be less precise and less efficient.

The plastic hardness values showed a similar pattern to that of the Martens hardness values. LT aligners (printed using SLA) had the highest initial value, which decreased after water storage to values almost identical to those of the PET-G reference material. In contrast, VP and TC aligners (printed using DLP) had a lower plastic hardness both before and after water storage, indicating potential limitations in force transmission in aligner applications.

Creep refers to the permanent yielding of a material under constant stress. Aligners with a tendency to creep may slowly flow or deform under prolonged pressure in the mouth. This can decrease the force transmitted to the teeth over time. Therefore, aligners need to have a low tendency to creep to maintain their dimensional stability and controlled force effect while being worn. The thermoplastic reference material (BL) showed the lowest creep tendency before and after water storage. In all materials, the creep tendency increased slightly after water storage. LT aligners showed high creep values initially, but these fell to the levels of the reference standard after water storage because of changes in mechanical properties. LT and BL had similar Martens hardness and plastic hardness values when stored in water, but still differed in indentation creep. The stable mechanical properties of BL aligners when exposed to moisture indicates greater long-term consistency in the oral environment.

The present results confirm observations from other studies examining the mechanical properties of 3D-printed materials. These findings emphasize that the sensitivity to artificial aging caused by exposure to water needs to be evaluated in 3D-printed materials before they are used. These findings also raise questions about the long-term usability of these aligners. However, they are typically worn for only 7–14 days, so their long-term stability may not actually be that important [25,31,32].

To date, only a few studies have compared the mechanical properties of directly printed aligners and thermoformable aligners. Most studies have used TC-85 because this was the first material to be approved for direct 3D printing of aligners and has received much attention because of its flexibility and viscoelastic properties. Shirey et al. investigated not only the modulus of elasticity but also the tensile strength and stress relaxation of different aligners (not TC-85), and showed that water storage has a significantly greater influence on the mechanical properties of additively manufactured aligners than on thermoformed aligners [21]. This contrasts with the findings of Can et al., who showed that the mechanical properties of printed aligners made from TC-85 were not affected by one week of wear [33]. Sayahpour et al. also investigated the mechanical properties of TC-85, thermoformable aligners made of thermoplastic polyurethane (TPU), and PET-G polymers after one week of use in an in vivo experiment. The TC-85 and unused TPU aligners had a similar hardness modulus and elastic index, but the restitution index (a measure of elastic rebound behavior) was significantly higher in TC-85 aligners, indicating greater force loss. The mechanical properties of the thermoformable aligners did not change after 7 days of intraoral use [34].

Another study compared the thermomechanical and viscoelastic properties of TC-85 aligners with those of PET-G aligners, including a U-shaped bending test to evaluate the shape-memory properties of both materials. TC-85 exerted an even, gentle force on the teeth because of its flexibility. This force decreased less significantly with repeated insertion, thereby maintaining a constant orthodontic effect [30].

### 4.3. Abrasion Resistance/Two-Body-Wear-Test

Abrasion is the mechanical material wear caused by chewing forces, cleaning with brushes, or tooth contact, and is a key parameter in terms of the long-term stress and durability of aligner materials. Abrasion was tested using a thermocycling process to match the fluctuating temperatures in the oral cavity during eating, drinking, speaking, and sleeping [35]. We based our approach on the test series conducted by Patzelt et al. to ensure a comprehensible and comparable methodology [36].

The SLA-printed material (LT) exhibited lower abrasion than the standard thermoformed material (BL) did. This higher resisance to abrasion in LT aligners is understandable considering the higher Martens hardness in LT aligners than BL aligners after water storage. Abrasion in the DLP-printed materials (VP, TC), could not be conclusively evaluated because of crack formation under load in the chewing simulator. A possible reason for this cracking is the lower initial Martens hardness values of these materials after water storage. This low hardness can lead to microcrack formation, which in turn promotes increased material fatigue and premature loss of function.

Only a few studies have measured abrasion because aligners are usually only worn for about 14 days, so abrasion is a rather minor issue. However, it is important to consider the release of microplastics through abrasion. Quinzi et al. investigated the release of microplastics from various PET-G and TPU thermoformed aligners after 7 days of simulated aging in artificial saliva under mechanical friction. They found microplastic particles between 5 and 20 μm in all aligners tested. Over 50% of particles found were in the range of 5–20 μm [37]. The advantage of using printed aligners in orthodontic treatment is that they are more abrasion-resistant and therefore release fewer microplastics.

### 4.4. Hygroscopic Volume Increase

Water is a low-molecular medium, so it can diffuse into plastics and act as a plasticizer molecule by weakening intermolecular bonds between polymer chains. This makes the chains more mobile, reducing their stiffness and hardness. The findings of this study have demonstrated that the water sorption of printed aligner materials significantly influences their mechanical properties. In addition, changes in dimensions induced by water sorption can impair the accuracy of aligner fitting.

In our study, test specimens were stored in water as an ageing process at 37 °C for two weeks. Prior to measurement, the test specimens were placed in distilled water at a temperature of 22 ± 2 °C for three hours. A lumen increase of more than 3% was observed in all groups. Of note, the SLA-printed LT samples were already 4.6% larger than the milled titanium reference sample before immersion in water. This suggests that LT has different dimensions to the milled titanium reference sample, possibly because of the manufacturing process and the composition of the resin. Another possible cause could be incomplete polymerization, which facilitates the diffusion of water molecules into the polymer network [21]. Of note, the printed aligners were significantly smaller than the milled reference body when dry. The increase in hygroscopic volume could not be tested in the BL reference material because suitable premanufactured disks were lacking.

In contrast to our findings, Ryokawa et al. showed that PET-G exhibits a linear expansion of 8.8% after 24 h of immersion in distilled water at 37 °C [38]. In another in vitro study, Ihssen et al. were able to show that thermocycled PET-G samples absorb 48% more water than those immersed in distilled water at 22 °C. The elasticity modules and tensile strengths were significantly lower at 37 °C than at 22 °C. These results illustrate that extreme temperature changes and water sorption can reduce the elasticity module of PET-G, thereby reducing orthodontic forces during the wearing period [39].

Šimunović et al., compared the water absorption, water release behavior, and diffusion kinetics of thermoformed aligners made of TPU and PET-G with those of 3D-printed aligners made of TC-85 [40]. The TPU material showed the fastest water absorption and also the fastest water release after removal from the water, while PET-G exhibited significantly slower moisture dynamics. The 3D-printed aligners absorbed less water overall, but released it again with a delay. This illustrates that water absorption and release by orthodontic aligners is significantly influenced by the material composition, the manufacturing method, and the layer structure of the materials [40].

Overall, the findings from this study and others show that water retention is an important parameter that influences dimensional stability and mechanical functionality during treatment.

### 4.5. Limitations

There were some limitations to this study. First, the test specimens (TC, VP) were provided by the manufacturers, so we can not confirm the adherence to the official protocols for VP and TC directly. Nevertheless, as the manufacturers assured strict compliance with the specified protocols, we assume that these requirements were implemented accordingly, although this could not be directly verified by us. This is particularly significant with regard to post-processing in 3D printing to ensure biocompatibility and the desired mechanical properties of the final material. Deviations could directly influence the material properties and thus the measurement results [41]. Second, we cannot rule out that the test specimens were affected (e.g., dried out) by the storage conditions prior to testing. This is reflected in the results of the indentation test and Martens hardness measurement, which were not normally distributed in VP aligners. However, these deviations could also have been caused by manufacturing-related factors.

A third limitation is that the LT material is currently not approved for the manufacture of aligners, but only for the production of occlusal splints and retention splints. However, we decided to include it in our investigation because several studies have shown that this material would be suitable for aligners [18,20,42]. Therefore, investigating LT as a potential aligner material appears reasonable, particularly to provide preliminary data on its mechanical behavior in comparison with established aligner materials.

A fourth limitation is that this was an in vitro study. Although the tests were conducted in accordance with the relevant ISO standards to ensure methodological comparability, the results cannot be fully applied to clinical situations because patient variability cannot be taken into account. In particular, the constant test conditions at 22 ± 2 °C cannot realistically reflect the dynamic and complex intraoral conditions. This limitation underscores the need for further clinical research to ensure the performance and stability of 3D-printed aligners in the oral environment.

## 5. Conclusions

Taking into account the limitations described above, we have drawn the following conclusions based on our findings:

1. Storing the printed test specimens in water significantly influences the mechanical properties by reducing the flexural modulus, Martens hardness, and plastic hardness.
2. The flexural modulus of the LT material was very similar to that of the reference material BL when water was added; this indicates that LT is a suitable material for aligner production. Prior water storage appears to benefit the mechanical properties of this material.
3. Mechanical abrasion of aligners during wear could release microplastic particles, posing potential risks. Further studies are needed to accurately assess the extent and clinical relevance of microplastic release.

## Figures and Tables

**Table 1:** Information on the tested aligner materials including manufacturer, chemical composition, indication stated by the manufacturer and production method of the specimens. DLP = digital light processing, PET-G = polyethylene terephthalate glycol.

Name (Group Code)	Manufacturer	Composition	Indication	Production Method	Batch Number
Biolon (BL)	Dreve Dentamid (Unna, Germany)	PET-G	Aligner & occlusal splints	Thermoplastic	3787603 3778615
Dental LT Clear V2 (LT)	Formlabs (Sommerville, MA, USA)	Acrylates and methylacrylates	Occlusal splints	Stereolitho-graphy	DC01220818-02
V Print Splint Comfort (VP)	VOCO (Cuxhaven, Germany)	Acrylates and methylacrylates	Occlusal splints	DLP printing	2312708 2241162
TC-85 DAC (TC)	Graphy (Seoul, Republic of Korea)	Acrylates and urethaneacrylate	Aligner splints	DLP printing	1-C1101C12

**Table 2:** Results of the indentation test. HM = Martens hardness, H_plast_ = Plastic hardness, C_IT_ = Indentation creep, WS = Testing after water storage; different capital letters indicate statistically significant differences (*p* < 0.05) between materials with the same water storage condition; different lower-case letters indicate statistically significant differences (*p* < 0.05) between different water storage conditions of the same material.

Material	HM [N/mm^2^]	HM-WS [N/mm^2^]	H_plast_ [N/mm^2^]	H_plast_-WS [N/mm^2^]	C_IT_ [ ]	C_IT_-WS [ ]
BL	92 ± 12 Ba	91 ± 13 Ba	222 ± 39 Aa	202 ± 44 Aa	0.08 ± 0.01 Da	0.09 ± 0.01 Da
LT	133 ± 16 Aa	113 ± 10 Ab	278 ± 53 Aa	201 ± 39 Ab	0.13 ± 0.02 Cb	0.15 ± 0.02 Ca
VP	64 ± 10 Da	25 ± 3 Cb	64 ± 10 Ba	25 ± 3 Bb	0.21 ± 0.02 Bb	0.25 ± 0.02 Ba
TC	76 ± 6 Ca	18 ± 2 Db	88 ± 10 Ba	12 ± 1 Cb	0.24 ± 0.02 Ab	0.29 ± 0.02 Aa

**Table 3:** Volume relative to the CNC-milled reference body before water storage and volume increase relative to the reference body after water storage. Different capital letters indicate statistically significant differences (*p* < 0.05).

Material	Volume Relative to the Reference Body Before Water Storage [%]	Volume Increase Relative to the Reference Body After Water Storage [%]
LT	104.6 ± 1.5 A	3.6 ± 1.1 A
VP	83.9 ± 2.1 B	3.1 ± 0.9 A
TC	85.4 ± 2 B	3.6 ± 1.3 A

## Data Availability

The data and results presented in the study are included in the article; further inquiries can be directed to the corresponding author.
